# Long noncoding RNA SH3PXD2A-AS1 promotes colorectal cancer progression by regulating p53-mediated gene transcription

**DOI:** 10.7150/ijbs.58422

**Published:** 2021-05-11

**Authors:** Pingfu Hou, Tian Lin, Sen Meng, Meilin Shi, Fang Chen, Tao Jiang, Zhongwei Li, Minle Li, Sufang Chu, Junnian Zheng, Jin Bai

**Affiliations:** 1 Cancer Institute, Xuzhou Medical University, Xuzhou, Jiangsu, China.; 2 Center of Clinical Oncology, The Affiliated Hospital of Xuzhou Medical University, Xuzhou, Jiangsu, China.; 3 Jiangsu Center for the Collaboration and Innovation of Cancer Biotherapy, Cancer Institute, Xuzhou Medical University, Xuzhou, Jiangsu, China.; 4 Department of General Surgery, The Affiliated Hospital of Xuzhou Medical University, Xuzhou, Jiangsu, China.

**Keywords:** LncRNA, SH3PXD2A-AS1, CRC, Metastasis, P53

## Abstract

Long non-coding RNAs (lncRNAs) play key roles in various human cancers. We aimed to determine the key lncRNAs mediating colorectal cancer (CRC) progression. We identified some lncRNAs aberrantly expressed in CRC tissues by using lncRNA microarrays and demonstrated that SH3PXD2A-AS1 was one of the most highly overexpressed lncRNAs in CRC. We further aimed to explore the roles and possible molecular mechanisms of SH3PXD2A-AS1 in CRC. RNA ISH revealed that SH3PXD2A-AS1 was overexpressed in CRC compared with adjacent normal colon tissues and indicated poor prognosis in CRC. Functional analyses showed that SH3PXD2A-AS1 enhanced cell proliferation, angiogenesis, and metastasis. Mechanistically, SH3PXD2A-AS1 can directly interact with p53 protein and regulate p53-mediated gene transcription in CRC. We provided mechanistic insights into the regulation of SH3PXD2A-AS1 on p53-mediated gene transcription and suggested its potential as a new prognostic biomarker and target for the clinical management of CRC.

## Introduction

Colorectal cancer (CRC) is a major lethal malignancy worldwide, the incidence rate of which has rapidly increased recently in China 1. Poor prognosis has been observed in patients with distant metastasis, which is a leading cause of death in patients with cancer. Approximately 50% of patients with CRC die from developing distant metastasis 2. Therefore, discovering novel metastasis promoters and suppressors is essential.

Long noncoding RNA (lncRNA) is a class of large transcripts (>200 nucleotides) with limited protein-coding potential. LncRNAs play a key role in both physiological and pathological processes, including cancer 3. The lncRNAs CCAL, CCAT1, FEZF1-AS1, PVT1 and DLEU1 are aberrantly expressed in CRC and regulate CRC tumorigenesis and progression 4-9. The lncRNAs DINO and MEG3 can also regulate p53 signaling 10, 11, suggesting that multiple lncRNAs can regulate p53. However, the functions and biological mechanisms of lncRNAs in the progression of CRC remain largely unknown.

In the present study, we identified lncRNAs aberrantly expressed in CRC tissues by using lncRNA microarrays. SH3PXD2A antisense RNA 1 (SH3PXD2A-AS1) was identified as one of the most upregulated lncRNAs in the lncRNA microarrays. Although it had been reported as a biomarker in colon and ovarian cancers 12, 13, the clinical roles and molecular mechanism of SH3PXD2A-AS1 should be fully addressed in CRC. We confirmed that SH3PXD2A-AS1 was upregulated in 70% CRC tissues and was associated with poor survival using tissue microarrays containing 568 CRC tissues and corresponding non-tumor adjacent tissues. Mechanistic studies firstly discovered that SH3PXD2A-AS1 promotes colorectal cancer progression by directly binding to p53 protein and regulating p53-mediated gene transcription in CRC. Functional analyses revealed that SH3PXD2A-AS1 enhanced cell proliferation, angiogenesis, and metastasis. These results highlighted a critical role for SH3PXD2A-AS1 in regulating CRC growth and metastasis through the p53 signaling pathway and suggest SH3PXD2A-AS1 as a new potential therapeutic target for CRC.

## Materials and Methods

### Cell lines and clinical samples

The CRC cell lines FHC, HCT116, DLD1, HT29, SW620, and SW480 were purchased from the cell bank of Chinese academy of sciences from 2014 to 2016. The cells were cultured following the instructions recommended by the cell bank. The cells were tested by short tandem repeat (STR) analysis and were validated to be mycoplasma-free.

CRC and matched adjacent tissues used for tissue microarray (TMA) construction and qRT-PCR were enrolled at Affiliated Hospital of Xuzhou Medical University from 2010 to 2015 in China. The TMAs slides included examination of 568 pairs of tissue specimens including 568 CRC and the matched corresponding adjacent normal colorectal tissues from CRC patient cohorts (As described in our previous work 14). Some of the tissues could not be used in the analyses steps as missing or bad conditions. The patient studies were conducted in accordance with Declaration of Helsinki. The use of these specimens and data for research purposes were granted approval by the Ethics Committee of the Affiliated Hospital of Xuzhou Medical University.

### LncRNA microarray analysis

Total RNA was extracted from five paired CRC tissues and NCTs using TRIzol Reagent (Invitrogen, USA). RNA quantity and quality were measured by NanoDrop ND-1000. RNA integrity was assessed by standard denaturing agarose gel electrophoresis. LncRNA microarray was performed by (Aksomics, Shanghai, China). Arraystar Human LncRNA Microarray V3.0 is used for detecting the global profiling of human LncRNAs. Agilent Feature Extraction software (version 11.0.1.1) was used to analyze acquired array images. LncRNAs were deemed differentially expressed if their fold change between the CRC and NCT (normal colon tissue) groups exceed 2.0 and their P-values were less than 0.05.

### RNA-sequence

RNA-seq was performed to detect the mRNA expression profiles of SH3PXD2A-AS1-silenced CRC cells at CapitalBio Technology (Beijing, China) using Illumina HiSeq sequencer (Illumina). DESeq2 was used to analyze the DEGs between samples. The differential genes were selected with fold change > 1.5. A GO functional enrichment analysis and KEGG pathway enrichment analysis were performed for the DGEs using the Goseq R package and KOBAS 3.0 software (Available online: http://kobas.cbi.pku.edu.cn). Gene Set Enrichment Analysis (GSEA) software 15 was used analyze the expression profiles to reveal the related biological pathways. GSEA2-2.2.4 software was used for gene set enrichment analysis of the RNA-seq data.

### Vector constructs and Stable cell lines construction

The SH3PXD2A-AS1 sequence (NR_038940.1) was synthesized by GENEWIZ Biotechnology (China) and cloned to the lentivirus vector pCDH1-CMV-MSC-EF1-GFP-Puro vector. The shRNA sequences of SH3PXD2A-AS1 was synthesized and cloned into the pLKO-Tet-On vector. The SH3PXD2A-AS1 overexpression/knockdown or the control vectors were co-transfected into HEK293T with the packing plasmids (psPAX and pMD2G) for producing viral particles using Lipofectamine 2000 (Invitrogen) as described before 16. And then, Stable cell lines overexpressing or lacking SH3PXD2A-AS1 were generated by infecting with lentivirus and selected with 2 µg/mL puromycin for 1-2 weeks. Doxycycline is used to induce shRNA expression, 20 ng/ml of Dox was used to induce SH3PXD2A-AS1 shRNA expression (48-72 hours) *in vitro* and 2 mg/ml of Dox administered in 5% drinking water was used to induce shRNA expression *in vivo*. The efficiency of SH3PXD2A-AS1 overexpression or knockdown was assessed using qRT-PCR. The shRNA sequences are listed as below:

- shSH3PXD2A-AS1#1 sense: CCGGGCAGCTCAGGTGTATGTAAGGCTCGAGCCTTACATACACCTGAGCTGCTTTTTG;
- shSH3PXD2A-AS1#2 sense: CCGGGCACCAAGAGAGCCCTAAAGACTCGAGTCTTTAGGGCTCTCTTGGTGCTTTTTG;

### RNA pull-down and Mass Spectrometry Analysis

The templates for *in vitro* transcription were cloned from SH3PXD2A-AS1 expression vector by PCR using primers containing a T7 promoter. Then, 500 ng DNA templates were used synthesize RNA using T7 RNA polymerase Transcription system (Promega) and Biotin RNA Labeling Mix (Roche), according to the manufacturer's instructions. The RNA pull-down assays were performed as previously described 17. For RNA pull-down assay using recombinant proteins, human TP53-6*His fusion protein (Ag16596, Proteintech Group, Wuhan, China) was used. Proteins obtained from RNA pull-down assay were used for running on SDS-PAGE gel. Protein bands were visualized by running on 10% SDS-PAGE followed by Coomassie brilliant blue staining. The protein bands were cut off for Mass Spectrometry analysis (Shanghai Applied Protein Technology Co., Ltd.) on Q Exactive^TM^ mass spectrometer (Thermo Fisher Scientific). For Flag-MS2bp-MS2bs-based RNA pull-down assay, SH3PXD2A-AS1 was cloned to the pCDNA3-12xMS2bs. Specifically, pcDNA3-FlagMS2bp and pcDNA3-SH3PXD2A-AS1-MS2bs were co-transfected to 293T cells, and the cells were harvested and performed RNA immunoprecipitation with anti-Flag antibody (Sigma) as previous described 18. The retrieved proteins were subjected to SDS-PAGE and detected by Western blots lastly.

### *In vitro* Transcription

The temples for *in vitro* transcription were cloned from SH3PXD2A-AS1 expression vector by PCR using primers containing a T7 promoter and purified using DNA Gel Extraction Kit. Full-length SH3PXD2A-AS1 cDNA temple was cloned using paired primers P1 and P8, SH3PXD2A-AS1 cDNA temple fragment 1-580 was cloned using paired primers P1 and P5, fragment 1-1077 was cloned using paired primers P1 and P6, fragment 1-1650 was cloned using paired primers P1 and P7, fragment 581-2023 was cloned using paired primers P2 and P8, fragment 1078-2023 was cloned using paired primers P3 and P8, fragment 1651-2023 was cloned using paired primers P4 and P8. Then, 500 ng DNA temples were used synthesize RNA using T7 RNA polymerase Transcription system (Promega) and Biotin RNA Labeling Mix (Roche), according to the manufacturer's instructions.

- P1: GATCACTAATACGACTCACTATATTCTAGAGCTAGCGAATTC;
- P2: GATCACTAATACGACTCACTATATTCACCAACTACCAACTATT;
- P3: GATCACTAATACGACTCACTATAAGACGTTCTCATGCCACCCT;
- P4: GATCACTAATACGACTCACTATAGGGGAGGCCAAGTGTCTGCAT;
- P5: GATGGCTTAAGTGAGACCTATGTGG;
- P6: AAAGCCTATGTCTGGAGTTA;
- P7: CAAGATCTGTAAGTCACCGT;
- P8: CATAGTTGTGTGACTCTGG.

### RNA *in situ* hybridization (ISH) and RNA fluorescence ISH (FISH)

The RNAscope® probe targeting SH3PXD2A-AS1 was designed and synthesized by Advanced Cell Diagnostics, and the expression of SH3PXD2A-AS1 was detected in TMAs and cells using the RNAscope® 2.5 HD Reagent Kit (Red) according to the manufacturer's instructions. The ISH score of SH3PXD2A-AS1 staining was semi-quantitative according to the manufacturer's instructions of RNAscope® 2.5 HD Reagent Kit (Red).

### Nuclear and cytoplasmic RNA separation

Nuclear and cytoplasmic RNA were purified by PARIS™ Kit (Thermo Fisher) followed the according to the manufacturer's instructions. The purified RNA was used as temple to generate cDNA using the HiScript 1^st^ Strand cDNA Synthesis Kit (Vazyme Biotech, Nanjing, China). Then, relative RNA expression was detected by q-RT-PCR as described above.

### Western blots

Western blots were performed as previously reported 19. Immunoblot assays were used to detect the protein levels by using primary antibodies Anti-GAPDH and Anti-p53 antibodies were purchased from Santa Cruz Biotechnology;; Anti-Nanog, Anti-PARP, Anti-Cleaved PARP, Anti-Cleaved Caspase-3, Anti-OCT4 and Anti-CD133 antibodies were purchased from Cell Signaling Technology company; Anti-CD44, Anti-VEGF, Anti-MET, Anti-PCNA, Anti-GDF15, Anti-SCD, Anti-CX3CL and Anti-FAS were purchased from Proteintech Group; Anti-HIF1α (Abcam).

### RNA extraction and quantitative real-time PCR (qRT-PCR) assay

Total RNA from cell and tissue samples was isolated using TRIzol Reagent (Invitrogen, USA) according to the manufacturer's protocols, and cDNA was synthesized using the HiScript 1^st^ Strand cDNA Synthesis Kit (Vazyme Biotech, Nanjing, China). Realtime PCR was carried out on ABI-7500 using UltraSYBR One Step RT-qPCR Kit (CWBIO, Beijing, China). The relative lncRNA and mRNA expression levels were normalized to 18s rRNA and GAPDH. The primers are listed in Supplemental Materials and Methods.

### Cell proliferation and apoptosis analyses

CCK-8 assay was applied to measure the cell proliferation according to the Cell Counting Kit-8 manufacturer's protocol (Dojindo). Annexin V-FITC/PI apoptosis detection kit was used to detect the apoptosis rates according to the manufacturer's protocol (KeyGEN BioTECH, Nanjing, China).

### Colony formation, Cell migration and invasion assay

The Colony formation, Cell migration and invasion assays were performed as described previously 19.

### Wound healing assay

Wound-healing assay was performed as previously described 18. In brief, cells were seeded at a density of 1×10^6^ cell/well onto six well plates. cell layers were wounded using a sterile 10 μl pipette tip after the cells adhering to the plate; the suspended cells were washed away with PBS, and then the cells were cultured in medium with 1% FBS. Gap areas were photographed with a light microscope at different time periods.

### Tube formation assay

Tube formation assay was performed following the protocol as described previously 20. The 96-well plate was coated with 50 μl matrigel ™ (BD Biosciences) and kept at 37 °C for 2 hours. 1 × 10^4^ HUVECs were suspended in 100 μl conditioned medium and seeded into the matrigel-coated 96-well plate and cultured for 4 hours, photos were taken under a microscope, and the complete tubular structures were counted.

### Mammosphere formation assay

Mammosphere formation assays were performed as previously reported 18. HCT116 and SW620 single-cell suspensions were plated in the ultralow attachment six-well plates (Corning) at a density of 10 000 cells/ml and grown in DMEM/F12 medium (serum free) supplemented with 20 ng/ml B27 (Invitrogen), 20 ng/ml EGF and 20 ng/ml bFGF (PeproTech) at 37 °C and 5% CO^2^. Fresh media was added every 3 days. Spheres >50 μm in diameter were counted at day 7.

### RNA immunoprecipitation (RIP)

The RIP experiments were carried out with the EZ-Magna RIP Kit (Millipore) according to the manufacturer's protocol. The primers using for quantitative RT-PCR analysis was listed in Supplementary Table S1.

### Chromatin immunoprecipitation (CHIP) assay

ChIP assay was performed according to the manufacturer's instructions of ChIP assay kit (Upstate Biotechnology, Lake Placid, NY). Briefly, the anti-p53 antibody immunoprecipitated DNA was obtained by eluting the Protein A/G beads with ChIP elution buffer, and was purified for analysis using the promoter-specific primers through qPCR. Meanwhile, anti-IgG antibody was used in CHIP assays as negative control. The primers sequences of the promoters were provided in Supplementary Table S1.

### Animal work

The female BALB/c nude mice (6-8 weeks old) were purchased from Beijing Vital River Laboratory Animal Technology Co., Ltd. (Beijing, China). The BALB/c nude mice were fed under specific pathogen-free condition. The animals were maintained in a controlled environment with controlled temperature (~25 °C), humidity (50 70%) and (light, 07:00; dark, 22:00). Specially, for the xenograft model, the total of 18 female mice were injected with control (1×10^6^) and shSH3PXD2A-AS1 control (1×10^6^) stable SW620 cells, and 2 mg/ml of Dox administered in 5% drinking water was used to induce shRNA expression. Seven days later, the subcutaneous tumors can be observed by vision. Tumors volumes were measured by multiplying long diameter and wide diameter every two days. The tumor volume was calculated using the formula V =a × (b × b)/2, where a is the largest and b is the smallest diameter. Lung metastasis model was established by tail vein injection. The total of 16 female BALB/c nude mice (6-8 weeks old) were randomly divided into three groups: control, SH3PXD2A-AS1 overexpression HCT116 cells. 1 × 10^6^ HCT116 cells suspended with 200 μL PBS were injected through tail vein. 8 weeks later, mice were sacrificed by cervical dislocation. The lungs of the mice were excised, and each lung was fixed by 4% paraformaldehyde for further H.E. staining and ISH assays and metastatic nodules on the surface of each lung were counted. All animal experiments were approved by the Animal Care Committee of the Xuzhou Medical University.

### Statistical analysis

Data were analyzed with SPSS 20.0 software (SPSS Inc., Chicago, IL, USA) and GraphPad Prism 7. The Student's t test, χ^2^ test, Chi-square test and log-rank test were used to determine the statistical significance of differences between groups. The Kaplan-Meier method and were used to estimate survival. p < 0.05 was considered statistically significant.

## Results

### SH3PXD2A-AS1 overexpression occurs in CRC and indicates poor prognosis

**Figure 1:**
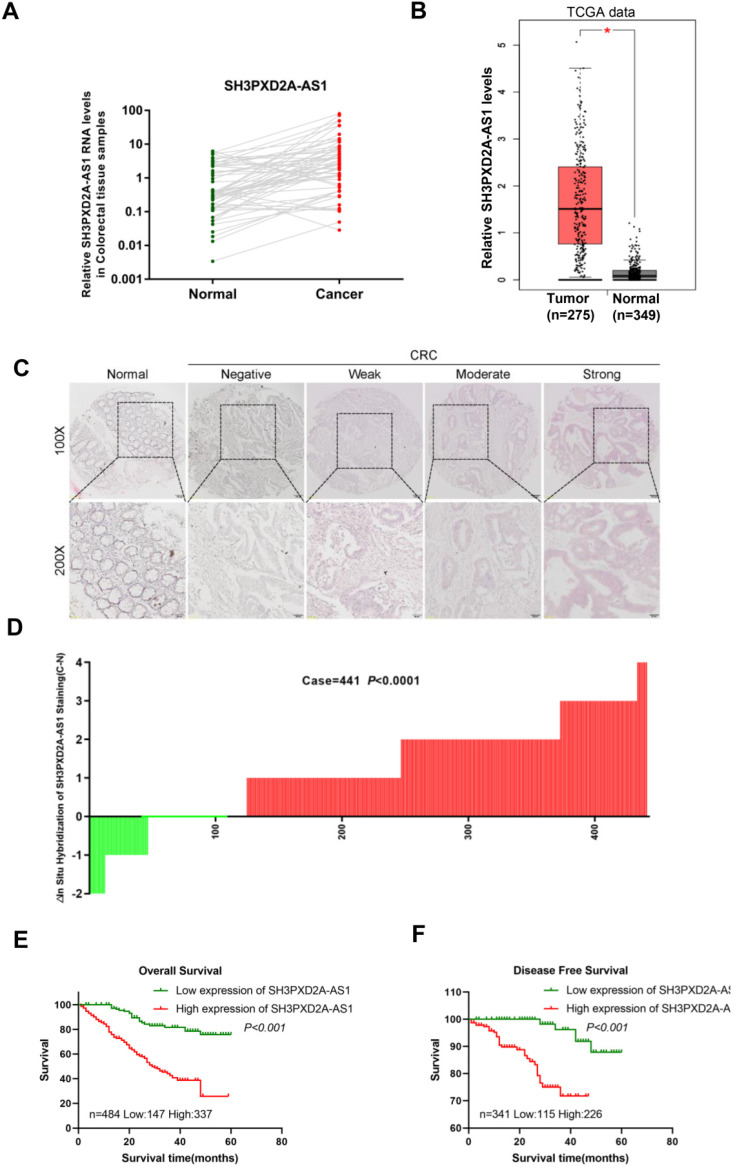
SH3PXD2A-AS1 is observed overexpression occurs in CRC and indicates poor prognosis. **(A)** Relative expression levels of SH3PXD2A-AS1 in paired CRC and NCTs were quantified by qRT-PCR (n=57, p = 0.0006). (**B**) Relative expression of SH3PXD2A-AS1 in normal and tumor tissues of colon by using TCGA data analyzed in the GEPIA website (http://gepia.cancer-pku.cn/index.html). **(C)** Representative images of SH3PXD2A-AS1 staining detected by SH3PXD2A-AS1 probes by using RNAscope® 2.5 HD Reagent Kit in CRC TMAs were shown. **(D)** Staining intensities of SH3PXD2A-AS1 in CRC compared with paired adjacent non-cancerous tissue. N, paired adjacent non-cancerous tissues. C, colorectal carcinoma tissues (p < 0.001). **(E-F)** Kaplan-Meier survival curves depicting overall survival (n = 484, p < 0.001) and disease free survival (n = 341, p < 0.001) of patients with CRC stratified by SH3PXD2A-AS1 staining in CRC tissues.

In our previous studies, LncRNA expression profiles were analyzed in paired CRC tissues and normal colorectal tissues (NCTs) to screen differentially expressed lncRNAs by using lncRNA microarray. We identified numerous differently expressed lncRNAs and SH3PXD2A-AS1 was one of the most expressed lncRNAs in the microarray 21. In this study, we focused on SH3PXD2A-AS1 for further studies. Expressions of SH3PXD2A-AS1 were validated in a small CRC cohort, revealing that SH3PXD2A-AS1 was upregulated in CRC tissues relative to adjacent NCTs, as shown in RT-PCR (p = 0.0006, Figure 1A) and gel electrophoresis results (Figure S1A). The GEPIA dataset 22 also revealed that SH3PXD2A-AS1 was up regulated in CRC relative to normal colon tissues (Figure 1B) and SH3PXD2A-AS1 expression was elevated in CRC with advanced tumor stage by using the TCGA data (Figure S1B). SH3PXD2A-AS1 expression was then examined by *in situ* hybridization (ISH; RNAscope®) in an expanded CRC cohort. The ISH arrays showed that SH3PXD2A-AS1 was overexpressed in CRC tissues relative to adjacent NCTs (n = 441, p < 0.001, Figure 1C-D). Furthermore, ISH analysis demonstrated that high SH3PXD2A-AS1expression was significantly upregulated in samples with advanced stage III/IV compared with stage I/II tumors. High SH3PXD2A-AS1expression was also associated with high differentiation (p = 0.001) and depth of invasion (p = 0.015, Table 1). Survival analysis also showed that high SH3PXD2A-AS1 expression was significantly correlated with poor overall survival (p < 0.0001, Figure 1E) and disease-free survival (p = 0.0002, Figure 1F). Finally, univariate analysis revealed that SH3PXD2A-AS1 was significantly associated with poor OS (hazard ratio [HR], 4.493; 95% confidence interval [CI], 2.845-7.097; p < 0.001), and DFS (HR, 7.780; 95% CI, 2.659-22.769; p < 0.001; Table 2). Multivariate analysis demonstrated that SH3PXD2A-AS1 expression was also an independent prognostic marker for both OS (HR, 3.833; 95% CI, 2.407-6.114; p < 0.001) and DFS (hazard ratio, 5.178; 95% CI, 1.711-15.68; p = 0.004; Table 3).

### SH3PXD2A-AS1 promotes CRC cell proliferation, migration, and invasion

**Figure 2:**
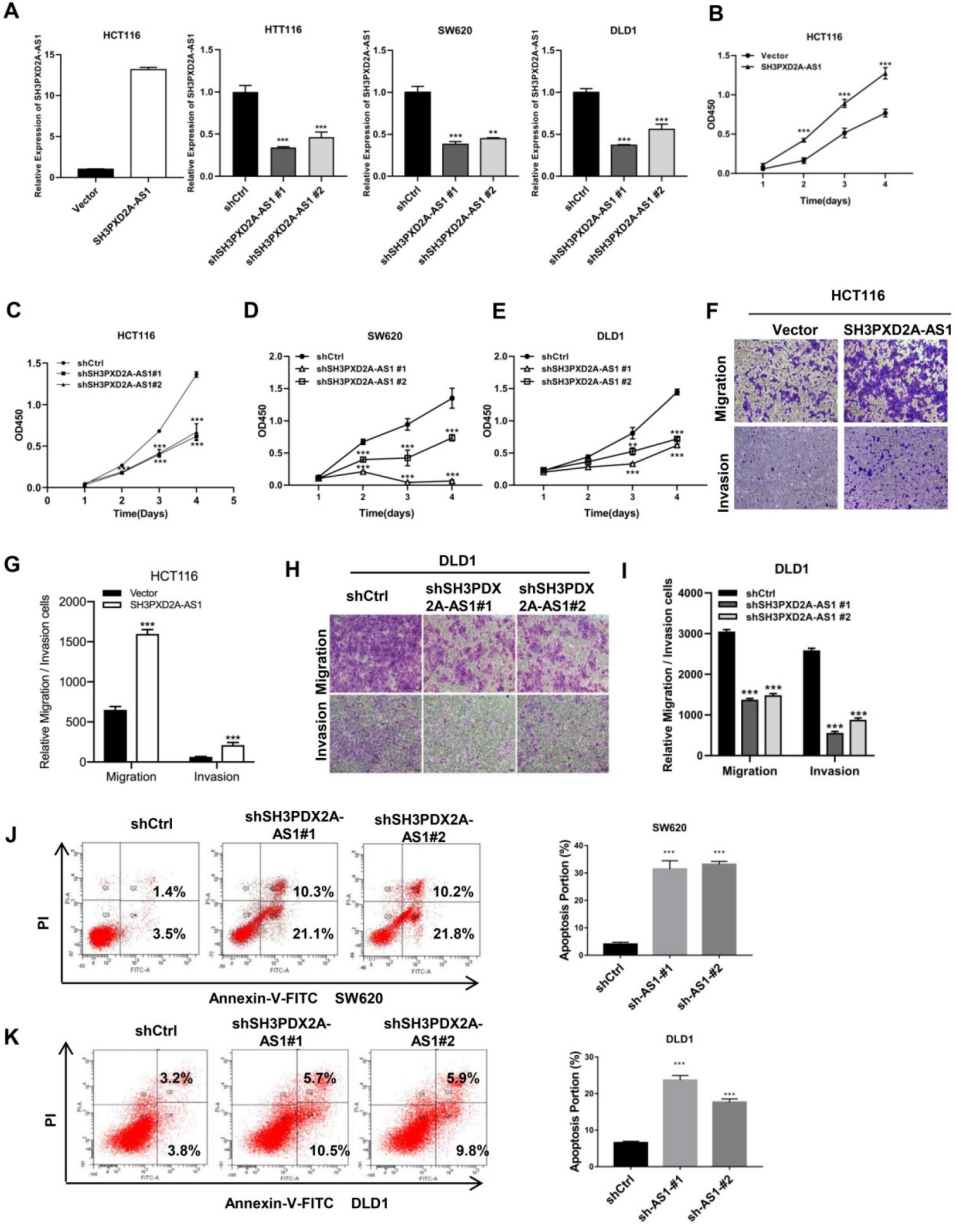
SH3PXD2A-AS1 promotes CRC cell proliferation, migration and invasion. **(A)** Relative expression of SH3PXD2A-AS1 in SH3PXD2A-AS1 stable overexpression or knockdown (Dox induced) CRC cell lines were quantified by qRT-PCR. Values were normalized against 18s rRNA from three independent experiments. **(B-E)** Effect of SH3PXD2A-AS1 overexpression or knockdown on CRC cells proliferation as assessed by Cell Counting Kit-8 (CCK8) assays. **(F-G)** Cell migration and invasion of HCT116 cells with SH3PXD2A-AS1 overexpression were measured as percent cells migrating to chambers. **(H-I)** Cell migration and invasion of DLD1 cells with SH3PXD2A-AS1 knockdown were measured as percent cells migrating to chambers.** (J-K)** Effect of SH3PXD2A-AS1 knockdown on SW620 and DLD1 cell apoptosis were assessed by Annexin V-FITC/PI staining. **p < 0.01, ***p < 0.001.

Endogenous SH3PXD2A-AS1 expression was measured in colorectal cell lines. Results showed that SH3PXD2A-AS1 expression was higher in CRC cells than in normal colon cells (Figure S2A). Subsequently, to investigate the biological role of SH3PXD2A-AS1 in CRC, we established stable knockdown or overexpression cell lines by using shRNAs or SH3PXD2A-AS1 overexpression lentivirus (Figure 2A). We then examined the effect of SH3PXD2A-AS1 on cell proliferation by CCK-8 assays. Results showed that SH3PXD2A-AS1 overexpression increased HCT116 cell proliferation (Figure 2B). Conversely, HCT116, SW620, and DLD1 cells depleted of SH3PXD2A-AS1 displayed reduced rates of cell growth compared with the corresponding controls (Figure 2C-E). Colony formation assays also suggested that SH3PXD2A-AS1 promotes tumor cell growth in colon cancer cells (Figure S2B-C). Furthermore, similar overexpression and silencing experiments revealed a positive role for SH3PXD2A-AS1 in promoting cell migration and invasion in CRC cells by using Transwell assays (Figure F-I). Wound healing assay also showed that SH3PXD2A-AS1 knockdown repressed cell mobility (Figure S2D). Finally, SH3PXD2A-AS1 knockdown caused cell apoptosis (Figure J-K) and increased cleaved casp3 and PARP expression levels, did not affect the expression of p53 (Figure S2E). In summary, these results suggest that SH3PXD2A-AS1 is essential in governing CRC cell proliferation, migration, and invasion.

### SH3PXD2A-AS1 governs the self-renewal maintenance of colorectal cancer stem cell and promotes CRC cell angiogenesis

**Figure 3:**
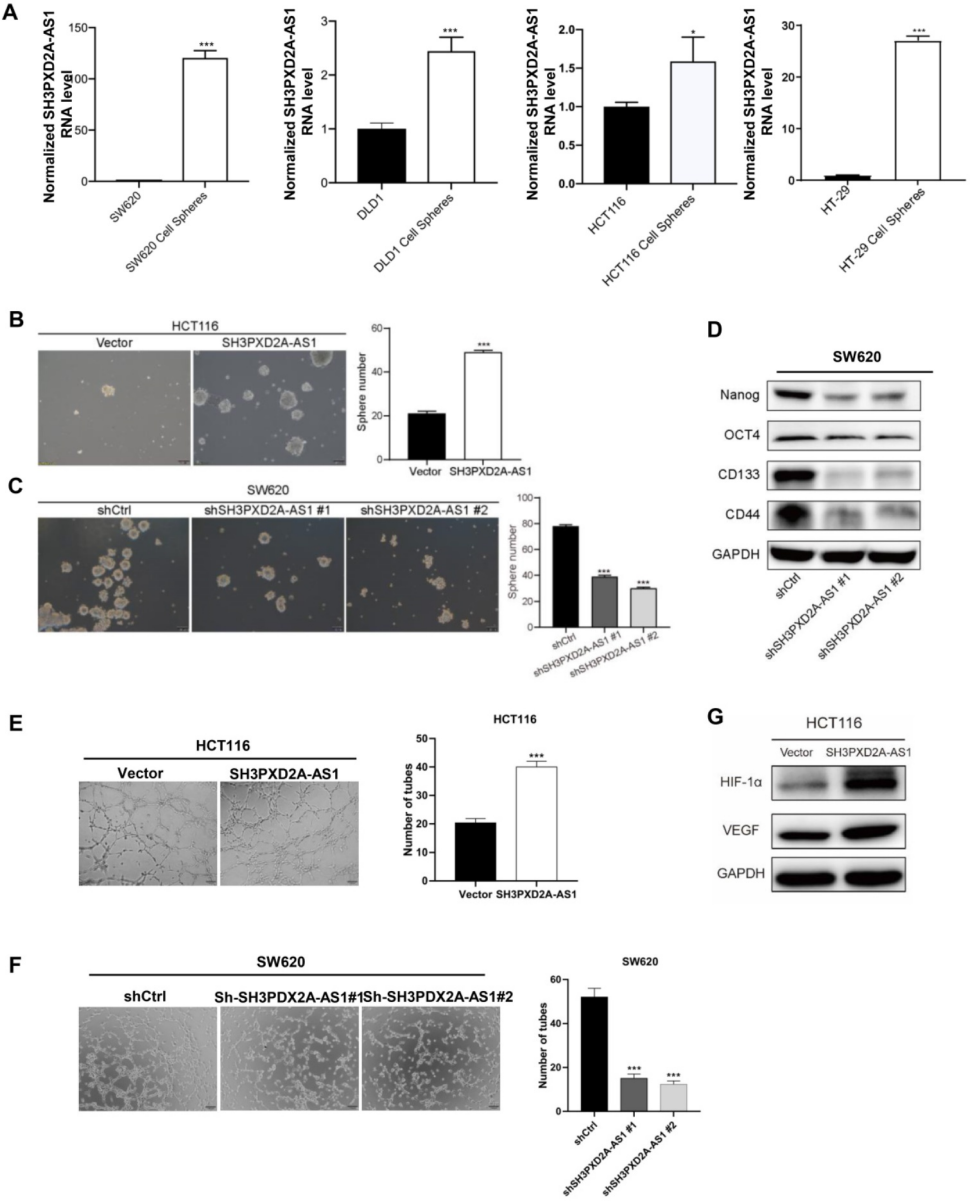
SH3PXD2A-AS1 maintains CRC stem cell properties and promotes cell angiogenesis.** (A)** Relative expression of SH3PXD2A-AS1 in adherent CRC cells and the corresponding suspension cultured tumor-spheres. Values were normalized against 18s rRNA from three independent experiments. **(B-C)** Effect of SH3PXD2A-AS1 overexpression or knockdown on mammosphere formation detected by mammosphere formation assays were quantified by sphere numbers. **(D)** Western blot of CSC markers: Nanog, OCT4, CD133 and CD44 in SH3PXD2A-AS1 knockdown SW620 cell. GAPDH was used as a loading control. **(E-F)** SH3PXD2A-AS1 positively regulated tube formation in CRC cells. The numbers of tubular structures formed by HUVECs were counted for in HCT116 and SW620 cells with SH3PXD2A-AS1 overexpression or knockdown. Data are presented as the means ± SD for experiments in triplicate. (G) Detection of HIF1α and VEGF protein expression in HCT116 cells with SH3PXD2A-AS1 overexpression. GAPDH was used as a loading control. Statistical analysis was performed by using two-tailed Student's. *p < 0.05, ***p < 0.001.

To identify whether SH3PXD2A-AS1 is involved in colorectal cancer stem cell (CSC), we cultured CRC cell lines in suspension cultured to acquire tumor spheres that may enrich CSCs. SH3PXD2A-AS1 levels were dramatically increased in tumor spheres compared with those in adherent colon cancer cells (Figure 3A). SH3PXD2A-AS1 overexpression increased the number of mammospheres formed (Figure 3B). Conversely, silencing SH3PXD2A-AS1 decreased the number of mammospheres (Figure 3C). Furthermore, SH3PXD2A-AS1 depletion significantly reduced the expression of the pluripotent transcription factors Nanog, Oct4, CD133, and CD44 compared with scrambled control (Figure 3D). This result suggested that SH3PXD2A-AS1 may mediate the self-renewal capacity of colorectal CSCs. Considering that angiogenesis is an important step for tumor growth and metastasis 23, we tested the effect of SH3PXD2A-AS1 on angiogenesis by tube formation assays. Results showed that the number of tubes formed by HUVECs cells was significantly increased and decreased in the conditioned medium collected from SH3PXD2A-AS1 overexpression and knockdown cells compared with the corresponding controls, respectively (Figure 3E-F). Additionally, the angiogenesis regulators HIF-1α and VEGF were significantly upregulated in HCT116 cells overexpressing SH3PXD2A-AS1 (Figure 3G).

### SH3PXD2A-AS1 directly interacts with p53 protein

**Figure 4:**
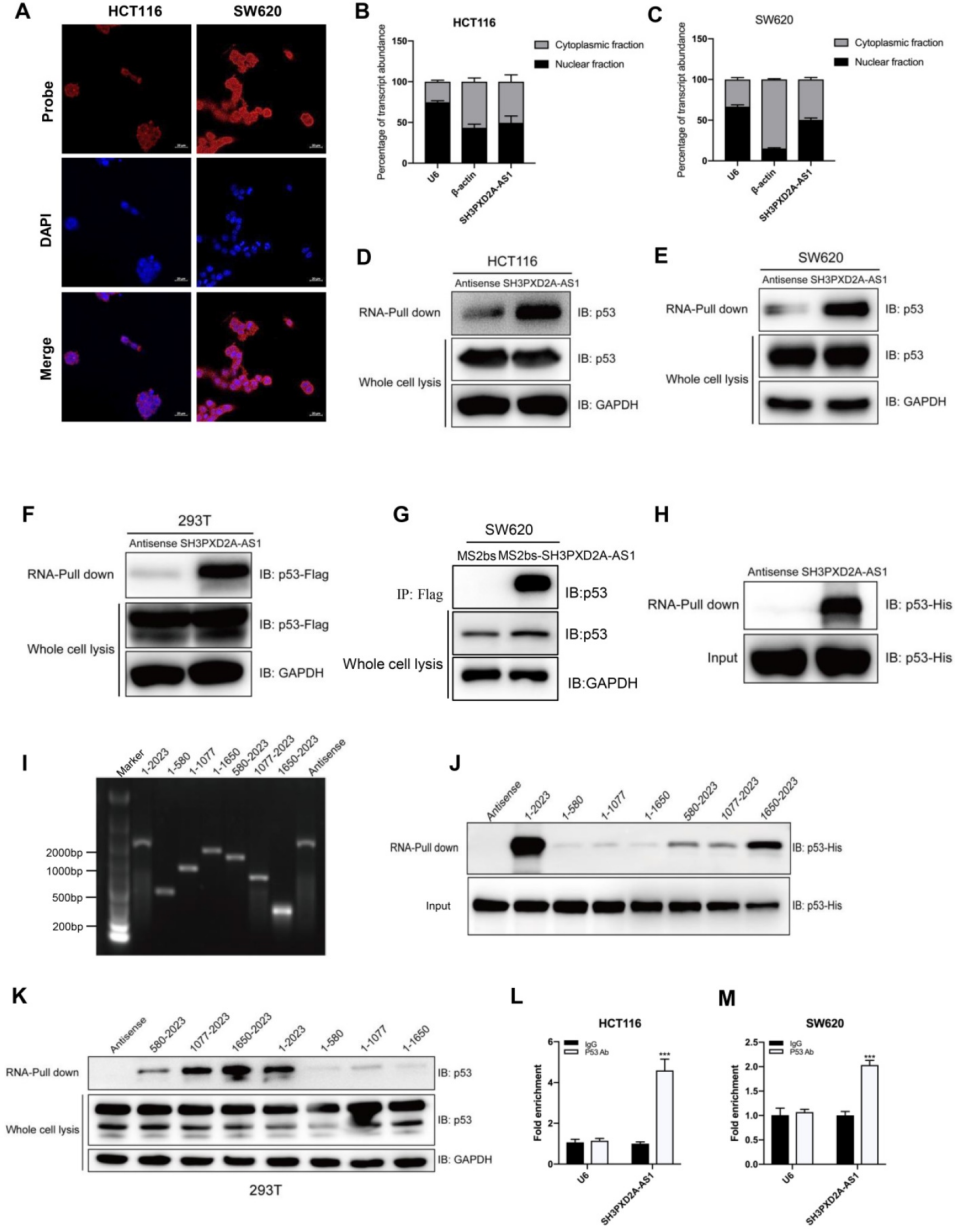
SH3PXD2A-AS1 directly interacts with p53 protein. **(A)** SH3PXD2A-AS1 intracellular localization was visualized in CRC cells by RNA-FISH assays. DAPI, 4', 6-diamidino-2-phenylindole. Probes, SH3PXD2A-AS1. Scale bar, 20 µm. **(B-C)** Fractionation of HCT116 and SW620 cells followed by quantitative real-time PCR. U6 RNA served as a positive control for nuclear gene expression. β-actin served as a positive control for cytoplasmic gene expression. **(D-E)** Biotin-RNA pull-downs were performed with HCT116 and SW620 cells by using full-length SH3PXD2A-AS1 transcript (sense), antisense. **(F)** Biotin-RNA pull-downs were performed in 293T cells transfected with wide type flag-p53 vector by using full-length SH3PXD2A-AS1 transcript (sense), antisense.** (G)** Flag-MS2bp-MS2bs-based RNA pull-down identified SH3PXD2A-AS1 interacted with p53 *in vivo*. Flag-MS2bp vector were co-transfected with 12×MS2bs or 12×MS2bs- SH3PXD2A-AS1 vector to 239T cells, then Flag-antibody were used to perform immunoprecipitation assay followed by detecting p53 protein. **(H)** Biotin-RNA pull-downs were performed by using recombinant p53-6*His fusion protein and full-length SH3PXD2A-AS1 transcript (sense), antisense. **(G)** Biotin-labeled SH3PXD2A-AS1 full-length and truncated fragments running in agarose electrophoresis gel. **(F)** Biotin-RNA pull-downs were performed by using p53-6*His fusion protein and SH3PXD2A-AS1 fragments. **(G)** Biotin-RNA pull-downs were performed by using 293T whole cell lysis and SH3PXD2A-AS1 fragments. **(H-I)** RIP assays were performed by using IgG and Anti-p53 antibody in HCT116 and SW620 cells followed by quantitative real-time PCR. U6 RNA served as a negative control. Data are shown as means ± SD. ***p < 0.001 by two-tailed Student's t test. Data are representative of at least three independent experiments.

To investigate the potential molecular mechanism of SH3PXD2A-AS1, we firstly tested the protein expression of Fish coded by SH3PXD2A gene. Results showed that SH3PXD2A-AS1 did not affect the expression of Fish (Figure S3A); it suggested that SH3APXD2A-AS1 might regulate biology functions through regulating other genes. Our RNA ISH experiments displayed that SH3PXD2A-AS1 localized both in the nucleus and cytoplasm of CRC tissues (Figure S3B). In addition, FISH (Figure 4A) and subcellular fractionation assays (Figure 4B-C) showed the cellular localization of SH3PXD2A-AS1 in CRC cells. Results showed that SH3PXD2A-AS1 localized both in the nucleus and cytoplasm in colon cancer cells. Furthermore, we performed RNA pull-down assay to identify SH3PXD2A-AS1-binding proteins. The retrieved proteins were subjected to SDS PAGE electrophoresis analysis, and the differential bands were selected for mass spectrum analysis. On the basis of the location and functions of proteins predicted by mass spectrum analysis, p53, a well-studied protein, was selected as an SH3PXD2A-AS1-associated protein (Figure S3C). RNA pull-down and Western blots assays further identified that SH3PXD2A-AS1 can interact with p53 in HCT116 (p53 wide type) and SW620 (p53 mutant) cells (Figure 4D-E). We also showed that SH3PXD2A-AS1 can interact with PARP by using RNA pull-down assay (Figure S3D-E). We further expressed exogenous wild-type Flag-p53 plasmid in HEK-293T cell. Western blots showed that SH3PXD2A-AS1 can also interact with Flag-p53 (Figure 4F). We then used a Flag-MS2bps-based RNA pull down assay to simulate endogenous combination between SH3PXD2A-AS1 and p53 protein. Results showed that SH3PXD2A-AS1 can also interact with p53 protein (Figure 4G). Furthermore, the purified fusion His-p53 protein was used in the RNA pull-down assay. Results showed that SH3PXD2A-AS1 can directly interact with p53 protein (Figure 4H). Subsequently, to identify which region of SH3PXD2A-AS1 binds to p53, we constructed a series of SH3PXD2A-AS1 deletion mutants. RNA fragments were *in vitro* transcribed and used for RNA pull-down assays. The Western blot analysis of p53 in protein samples pulled down by these different SH3PXD2A-AS1 fragments showed that the 1,650 to 2,023 deletion almost caused the complete loss of their ability to bind p53 (Figure 4I-K), suggesting that the region is essential for SH3PXD2A-AS1 binding to p53. RIP assays performed with an anti-p53 antibody demonstrated the association between p53 and SH3PXD2A-AS1 (Figure 4L-M). We also showed that SH3PXD2A-AS1 knockdown did not affect the expression of p53 (Figure S2E) and the distribution of p53 in nucleus or cytoplasm (Figure S4). These results indicate that SH3PXD2A-AS1 directly interacts with p53 protein.

### SH3PXD2A-AS1 regulates p53 target genes

**Figure 5:**
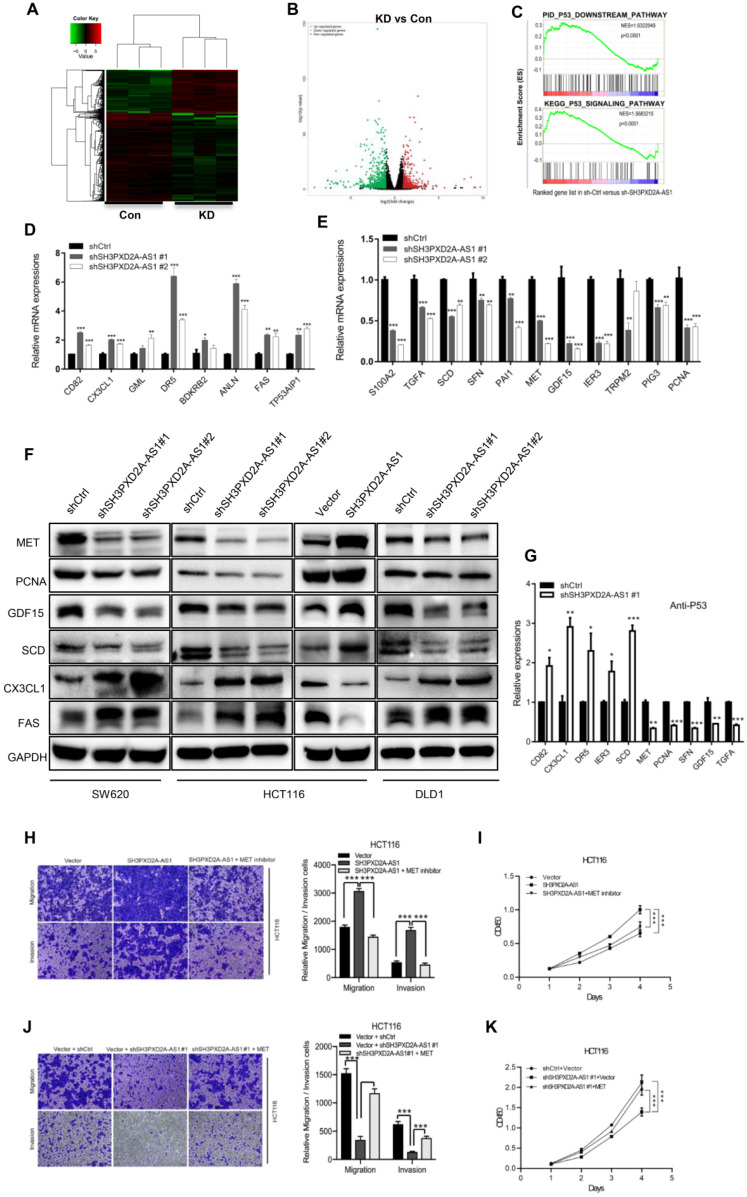
SH3PXD2A-AS1 regulates p53 target genes. **(A-B)** Differentially expressed genes that clustered by cohort and volcano plot. RNA-seq was taken in SH3PXD2A-AS1 knockdown (shRNA#1) SW620 cells. **(C)** GSEA analysis of SH3PXD2A-AS1-regulated genes revealed an enrichment of p53 downstream pathway and p53 signaling pathway in SH3PXD2A-AS1 knockdown SW620 cells.** (D-E)** Relative mRNA levels of p53 target genes those increased or decreased in SH3PXD2A-AS1 knockdown SW620 cells detected by quantitative real-time PCR. Values were normalized against 18s rRNA from three independent experiments. **(F)** Relative protein levels of several p53 target genes detected by Western blot in CRC cells with SH3PXD2A-AS1 overexpression or knockdown. GAPDH was used as a loading control.** (G)** Relative p53 binding ability on the promoters of its target genes detected by ChIP assay followed by quantitative real-time PCR in SW620 cells. (H-I) MET inhibitor (SU11274, 2.5μM was used) blocked SH3PXD2A-AS1 overexpression induced cell migration, invasion and proliferation in HCT116. (J-K) Re-expression of MET by using c-MET vector in SH3PXD2A-AS1 knockdown SW620 cells partly reversed the inhibition of cell migration, invasion and proliferation. Data are shown as means ± SD. Data are representative of at least three independent experiments. *p < 0.05, **p<0.01 and ***p < 0.001 by two-tailed Student's t test.

To evaluate the genes regulated by SH3PXD2A-AS1 in colon cancer, we performed RNA-seq to discover the differentially expressed genes regulated by SH3PXD2A-AS1 knockdown. A list of significantly differentially expressed genes that clustered by cohort and volcano plot is revealed (Figure 5A-B). The differentially expressed genes were defined by functionally enriched gene ontology (GO) terms and pathways involved in cadherin signaling pathway, Wnt signaling pathway, and P53 pathway (Figure S5A-B). Strikingly, gene set enrichment analysis (GSEA) also revealed that two published p53 pathway signatures were significantly enriched in SH3PXD2A-AS1-positive cells, strongly suggesting that SH3PXD2A-AS1 affects the expression of a subset of p53 target genes (Figure 5C). We hypothesized that SH3PXD2A-AS1 may affect p53 transcription. To verify the hypothesis, we further used RT-PCR to test numbers of known p53 transcriptional regulated target genes in the SH3PXD2A-AS1 knockdown or overexpression cells. The target genes were obtained from the p53 knowledgebase website (http://p53.bii.a-star.edu.sg/). Results showed that some known p53 target genes were upregulated (Figure 5D), downregulated (Figure 5E), and remained unchanged (Figure S6A) in SH3PXD2A-AS1 knockdown SW620 cells. Among the differential expressed genes, several downregulated genes, such as S100A2 24, TGFA 25, MET 26, PCNA 27, GDF15 28, SCD 29, and PAI1 30, have been reported as oncogenes. Some upregulated genes, such as CX3CL1 31, TP53AIP1 32, Fas 33, and DR5 34, were reported as tumor suppressors. Furthermore, we further verified the expression levels of several p53 target genes in the tumors formed by the SH3PXD2A-AS1 knockdown cells (Figure S6B) or in SH3PXD2A-AS1 overexpression HT29 cells (Figure S6C-D). The expression levels were consistent with the above tests. Western blot assays also suggested SH3PXD2A-AS1 regulated the protein levels of several p53 target genes (Figure 5F). Finally, we confirmed that SH3PXD2A-AS1 can affect the occupation ability of p53 to the promoter of p53 target genes through chromatin immunoprecipitation assays (Figure 5G). These results indicated that SH3PXD2A-AS1 interacts with p53 protein and affects p53 target gene transcription.

To evaluate whether the role of SH3PXD2A-AS1 was dependent on the expression of p53 in colon cancer, we further measured the functions of SH3PXD2A-AS1 in p53^-/-^ HCT116 cells. Results showed that SH3PXD2A-AS1 overexpression can promote cell migration and invasion in p53^-/-^ HCT116 cells, but the migration and invasion abilities were lower than that in p53+/+ HCT116 cells (Figure S7A-B). SH3PXD2A-AS1 knockdown also repressed the migration and invasion abilities, although not lower than that in p53+/+ HCT116 cells (Figure S7C-D). Results suggested that the functions of SH3PXD2A-AS1 were not completely dependent on p53 protein. Some other co-factors were involved in regulating the roles of SH3PXD2A-AS1.

The MET receptor tyrosine kinase is upregulated in various tumors, including CRC 35. MET is important in cell migration, invasion, and proliferation and correlates with prognostic parameters and poor survival in CRC 36, 37. Many inhibitors have been developed to target MET in the clinical trials 38. In the current study, MET expression was positively regulated by SH3PXD2A-AS1 in colon cancer cells, and MET is a downstream target gene transcriptionally activated by p53 39, 40. To assess the requirement for MET in SH3PXD2A-AS1-induced CRC malignant characteristics, we inhibited MET expression by MET inhibitor (SU11274) in HCT116 cells overexpressing SH3PXD2A-AS1 or increased MET expression by using MET plasmid in SH3PXD2A-AS1 knockdown HCT116 cells. MET inhibition markedly suppressed the effects of SH3PXD2A-AS1 overexpression on cell migration, invasion and proliferation (Figure 5H-I). MET overexpression markedly restored the cell migration, invasion and proliferation abilities repressed by SH3PXD2A-AS1 knockdown (Figure 5J-K). Finally, to determine the correlation between the expression of SH3PXD2A-AS1 and MET, we analyzed gene expression data from CRC tissue samples that are publicly available in GEO database (GSE14333) containing 290 CRC tissues 41. Results showed that the mRNA level of SH3PXD2A-AS1 positively correlated with MET in the CRC tissues (Figure S8). Thus, MET may play an important role in SH3PXD2A-AS1 induced migration and invasion in CRC.

### SH3PXD2A-AS1 enhances CRC tumor growth and metastasis *in vivo*

**Figure 6:**
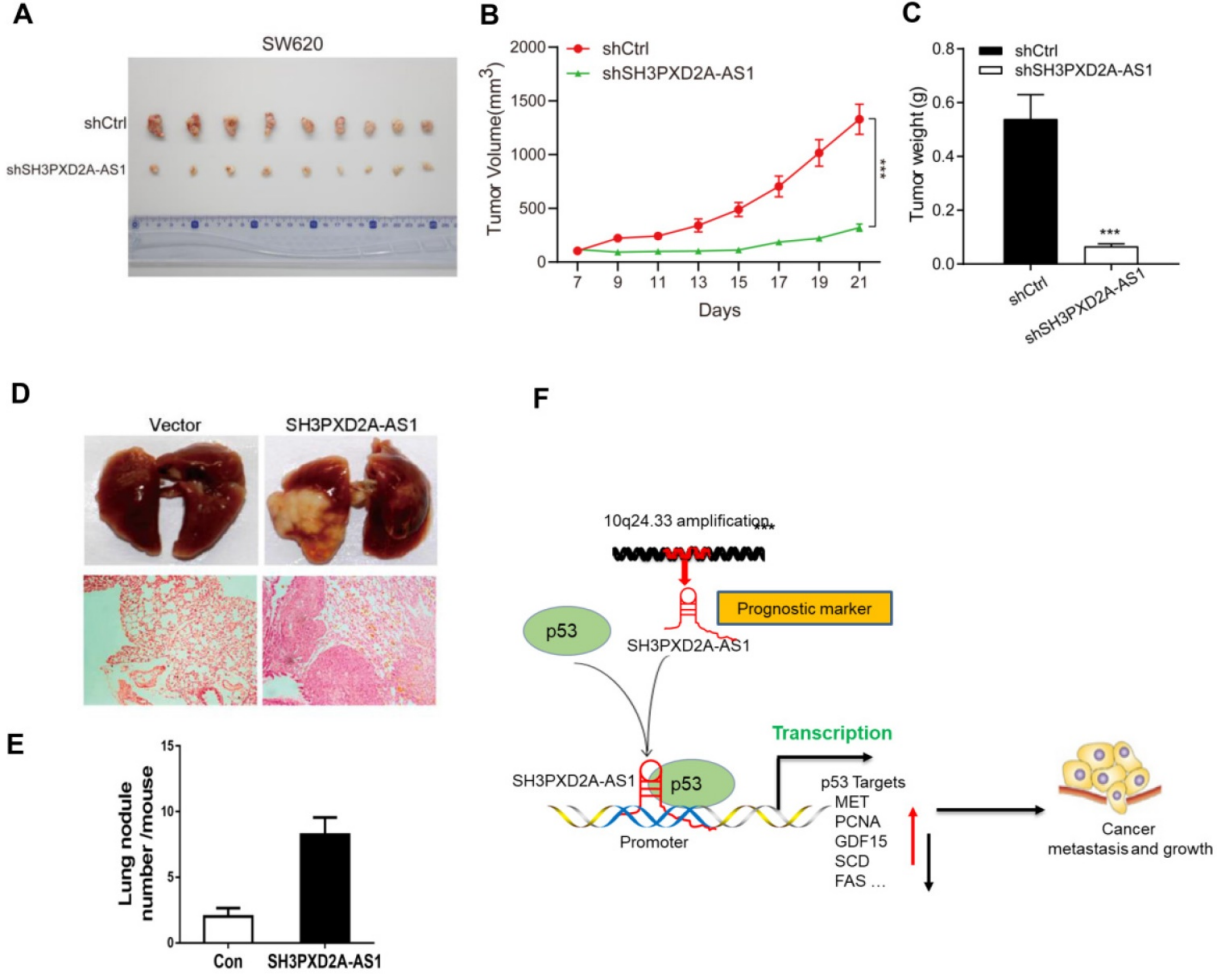
SH3PXD2A-AS1 promotes tumor growth and metastasis *in vivo*. **(A)** Stable SH3PXD2A-AS1 knockdown and control SW620 cells were subcutaneously injected into BALB/c female nude mice (n = 9, for each experimental group), and 2 mg/ml of Dox administered in 5% drinking water was used to induce shRNA expression. 1 × 10^6^ cells and Matrigel (Corning; 1:1 ratio) were subcutaneously injected into each mouse. Three weeks later, the xenograft tumors were peeled off and photographed. **(B-C)** Effect of SH3PXD2A-AS1 knockdown on the xenograft model was assessed by evaluating tumor volume and tumor weight. ***P<0.001** (D)** SH3PXD2A-AS1 overexpression HCT116 cells were injected via the lateral tail veins. Representative lung images at week 8, corresponding hematoxylin-eosin-stained lung sections are shown. **(E)** Lung nodules were analyzed as the numbers of nodules per mouse. Statistical analysis was performed by using two-tailed Student's t test. ***P<0.001.** (F)** A cartoon summarizing our findings. SH3PXD2A-AS1 interacts with p53 protein and regulated p53 mediated gene transcription, thereby promoting CRC growth, and metastasis.

To investigate the role of SH3PXD2A-AS1 in CRC tumor growth *in vivo*, we subcutaneously injected SH3PXD2A-AS1 knockdown or control cells into nude mice. SH3PXD2A-AS1 knockdown significantly decreased tumor growth *in vivo* compared with shRNA controls (Figure 6A-C). We then evaluated the effects of SH3PXD2A-AS1 on colon cancer cell lung metastasis by the tail vein injection model. In a typical procedure, HCT116 cells with SH3PXD2A-AS1 overexpression or control cells were injected to 8-week-old nude mice via their lateral tail vein. The mice were then executed to analyze lung metastasis status after 8 weeks. As shown in Figure 6D and 6E, multiple large metastatic foci were found in the lungs of control SH3PXD2A-AS1 overexpression group mice, and few were observed in the control group. In summary, these data indicate that SH3PXD2A-AS1 plays an important role in CRC tumor growth and metastasis *in vivo*.

In conclusion, our study reveals SH3PXD2A-AS1 directly interacted with p53 protein and regulated p53-mediated gene transcription in CRC, thereby promoting CRC growth and metastasis (Fig. 6F).

## Discussion

LncRNAs play an important role in various human cancers, but the engagement and contribution of lncRNAs remain insufficiently characterized. Although thousands of lncRNAs have been discovered to date, the possible role of lncRNAs in the regulation of cancer-related processes remains largely unknown. In this study, we identified a series of differentially expressed lncRNAs in CRC, including FEZF1-AS1, CCAT1, MIR17HG, and H19, which are potential oncogenes in colon cancer 5, 7, 42, 43. Of these lncRNAs, SH3PXD2A-AS1, a top overexpressed lncRNA in CRC, is a newly identified oncogenic lncRNA in human colorectal and ovarian cancer 12, 13. However, the detailed role and molecular mechanisms of SH3PXD2A-AS1 remain to be clarified.

The 2023 nt-long SH3PXD2A-AS1 gene is located on the opposite strand of SH3PXD2A on chromosome 10. SH3PXD2A-AS1 acts as an oncogene through regulating p57 and KLF2 in CRC 12. SH3PXD2A-AS1 is also correlated with overall survival in ovarian cancer 13. Although Ma et al. 12 suggested that SH3PXD2A-AS1 is overexpressed in CRC, they used a very small CRC cohort. In the current study, a CRC cohort containing 484 pairs of CRC tissue and adjacent NCTs were used to measure SH3PXD2A-AS1 expression. RNA Scope ISH assay revealed that SH3PXD2A-AS1 was overexpressed in CRC tissues (Figure 1D). Results also showed that SH3PXD2A-AS1 was positively associated with poor OS and DFS (Figure 1E-F). These results suggested that SH3PXD2A-AS1 expression can be used as a prognostic biomarker in CRC.

LncRNAs exert their functions via diverse mechanisms, including co-transcriptional regulation, modulation of gene expression, scaffolding of molecular complexes, pairing with other RNAs, and coding short peptides 44, 45. Here, we found that SH3PXD2A-AS1 directly interacts with p53 protein and regulates p53 target gene transcription to control CRC progression. Although numerous lncRNAs have been reported to be associated with the p53 regulatory pathway 46, functional lncRNAs binding to p53 have been rarely found. Damage-induced noncoding (DINO) lncRNA is the first well-known lncRNA that interacts with p53 protein and promotes p53 stabilization 10. In the current study, GSEA revealed that two published p53 pathway signatures were significantly enriched in SH3PXD2A-AS1-positive cells (Figure 5C). A series of p53 transcriptional regulated target genes, such as S100A2, TGFA, MET, PCNA, SCD, GDF15, PAI1, CX3CL1, TP53AIP1, FAS, and DR5, were affected by SH3PXD2A-AS1 (Figure 5D-E). S100A2, TGFA, MET, PCNA, SCD, GDF15, and PAI1 act as oncogenes by promoting cancer metastasis or proliferation 24-30. CX3CL1 is TP53AIP1, and Fas and DR5 act as tumor suppressors by promoting cell apoptosis or inhibiting cell migration 31-34. In the current study, the mRNA and protein levels of MET, PCNA, GDF15, and SCD were repressed, whereas those of CX3CL1 and FAS were increased in SH3PXD2A-AS1 knockdown cells (Figure 5D-E). This effect is consistent with the functions of SH3PXD2A-AS1 caused in CRC, such as in promoting tumor growth and metastasis. p53 acts as a repressor for SCD and IER3 transcription and activates the transcription of CD82, CXCL1, DR5, MET, TGFA, SFN, PCNA, and GDF15 (p53 knowledgebase: http://p53.bii.a-star.edu.sg/). Our CHIP assay showed that SH3PXD2A-AS1 knockdown enhanced the binding of p53 to the promoters of CD82, CXCL1, DR5, SCD, and IER3 and decreased the binding of p53 to the promoters of MET, TGFA, SFN, PCNA, and GDF15 (Figure 5G). The results of CHIP assays were consistent with the mRNA and protein levels of p53 target genes detected above. We also found the two oncogenes ANLN 47 and BDKRB 48, which showed upregulated expression in SH3PXD2A-AS1 knockdown cells (Figure 5D), which was not consistent with the role of SH3PXD2A-AS1 in CRC. This finding suggested that the role of SH3PXD2A-AS1 in regulating p53-medicated gene transcription is a very complicated event, and additional efforts should be made to identify the regulatory mechanisms. In this study, we focused the functions of MET in SH3PXD2A-AS1 regulated CRC biological progression and showed that MET is a target gene of SH3PXD2A-AS1 in CRC. The other target genes of SH3PXD2A-AS1 were not investigated in our present study, but we can see that they play roles in various aspects of biological progression. For example, GDF15 could also regulate metabolic diseases 49. We also found that SH3PXD2A-AS1 could interact with PARP, but the role of the interaction was not investigated. It suggested that the roles of SH3PXD2A-AS1 would also be very complicated and more effort should be made to uncover its functions.

SH3PXD2A-AS1's interaction can regulate p53-mediated gene transcription, suggesting a possible broad function for RNAs in the regulation of p53 signaling. The lncRNAs DINO and MEG3 can also regulate p53 signaling 10, 11, suggesting that multiple lncRNAs can regulate p53. Additionally, lncRNAs are expressed in a highly cell and tissue-specific manner 50. Thus, they may provide cells with unique opportunities to confer specificity on various aspects of cell biology.

In summary, this study provided novel evidence that SH3PXD2A-AS1 was upregulated in CRC and associated with poor prognosis. SH3PXD2A-AS1 enhanced CRC cell proliferation, angiogenesis, and metastasis. Mechanistic studies revealed that SH3PXD2A-AS1 directly interacted with p53 protein and regulated p53-mediated gene transcription in CRC. These results highlighted a critical role for SH3PXD2A-AS1 in regulating CRC growth and metastasis through the p53 signaling pathway and suggested that SH3PXD2A-AS1 may serve as a clinical diagnostic and prognostic biomarker and as a potential therapeutic target in CRC.

## Supplementary Material

Supplementary figures and tables.

## Figures and Tables

**Table 1:** Relationship between LncRNA SH3PXD2A-AS1 expression and clinicopathological features of CRC patients

Variables	All patients	SH3PXD2A-AS1 expression		P^*^-value
		Low (%)	High (%)	
All cases	484	147 (30)	337 (70)	
**Age**				0.002
<60 years	190	73 (38)	117 (62)	
≥60 years	294	74 (25)	220 (75)	
**Gender**				0.637
Males	282	88 (31)	194 (69)	
Females	202	59 (29)	143 (71)	
**TNM stage**				<0.001
I/II	281	105 (37)	176 (63)	
III/IV	203	42 (21)	161 (79)	
**Lymph node metastasis**				0.428
N0	310	98 (32)	212 (68)	
N1/N2/N3	174	49 (28)	125 (72)	
**Metastasis**				0.037
M0	455	143 (31)	312 (69)	
M1	30	4 (13)	26 (87)	
**Tumor diameter**				0.048
≤4.5 cm	247	65 (26)	182 (74)	
>4.5 cm	237	82 (35)	155 (65)	
**Differentiation**				0.001
Poor	414	114 (28)	300 (72)	
Moderate/High	70	33 (47)	37 (53)	
**Depth of invasion**				
T1/T2	102	41 (40)	61 (60)	0.015
T3/T4	382	106 (28)	276 (72)	

P^*^-value measured by Pearson's Chi-Squared test.

**Table 2:** Univariate Cox regression analysis of LncRNA SH3PXD2A-AS1 expression and clinicopathologic variables predicting the survival of CRC patients

Variables*	Overall survival		Disease-specific survival	
	HR (95%CI)	*P*	HR (95%CI)	*P*
SH3PXD2A-AS1	4.493 (2.845-7.097)	<0.001	7.780 (2.659-22.769)	<0.001
Age	1.342 (0.961-1.872)	0.084	1.949 (0.960-3.957)	0.065
Tumor diameter	1.702 (1.228-2.358)	0.001	1.660 (0.869-3.170)	0.125
Differentiation	0.736 (0.463-1.169)	0.194	0.587 (0.227-1.518)	0.272
Depth of invasion	1.805 (1.138-2.863)	0.012	3.028 (1.068-8.583)	0.037
TNM stage	2.668 (1.926-3.696)	<0.001	4.193 (2.126-8.271)	<0.001
LNM	1.361 (0.985-1.879)	0.062	1.692 (0.882-3.247)	0.114
Metastasis	4.166 (2.471-7.023)	<0.001		

Abbreviations: HR: Hazard Ratio; CI: Confidence Interval; LNM: Lymph Node Metastasis. Variables*: SH3PXD2A-AS1: Low *vs* High; Age: ≤60 (years) *vs* >60(years); Gender: Male* vs* Female; Tumor diameter: ≤4.5 (cm) *vs* >4.5 (cm); Differentiation: Poor *vs* Moderate/High; Depth of invasion: T1/T2 *vs* T3/T4; TNM stage: I/II *vs* III/IV; LNM: N0 *vs* N1/N2/N3; Metastasis: M0 *vs* M1.

**Table 3:** Multivariate Cox regression analysis of LncRNA SH3PXD2A-AS1 expression and clinicopathologic variables predicting the survival of CRC patients

Variable*	Overall survival		Disease-specific survival	
	HR (95%CI)	*P*	HR (95%CI)	*P*
SH3PXD2A-AS1	3.833 (2.403-6.114)	<0.001	5.178 (1.711-15.68)	0.004
Age	1.157 (0.825-1.622)	0.398	1.461 (0.708-3.015)	0.305
Differentiation	1.018 (0.638-1.622)	0.942	1.253 (0.480-3.275)	0.645
Depth of invasion	1.166 (0.720-1.886)	0.532	1.681 (0.575-4.916)	0.343
TNM stage	2.247 (1.599-3.159)	<0.001	2.990 (1.480-6.040)	0.002

Abbreviations: HR: Hazard Ratio; CI: Confidence Interval. Variables*: SH3PXD2A-AS1: Low *vs* High; Age: ≤60 (years) *vs* >60 (years); Differentiation: Poor *vs* Moderate/High; Depth of invasion: T1/T2 *vs* T3/T4; TNM stage: I/II *vs* III/IV; LNM: N0 *vs* N1/N2/N3.

## Abbreviations

lncRNA: long non-coding RNAs
CRC: colorectal cancer
ISH: *in situ* hybridization
FISH: fluorescence *in situ* hybridization
SH3PXD2A-AS1: SH3PXD2A antisense RNA 1
TMA: tissue microarray
NCT: normal colon tissue
GSEA: Gene Set Enrichment Analysis
CSC: cancer stem cell
RIP: RNA immunoprecipitation
CHIP: Chromatin immunoprecipitation
HIF-1α: Hypoxia-inducible transcription factors 1α
VEGF: Vascular endothelial growth factor
