# Supplementary material for: Long noncoding RNA SH3PXD2A-AS1 promotes colorectal cancer progression by regulating p53-mediated gene transcription
Source: Int J Biol Sci. 2021 May 11;17(8):1979–94. doi: 10.7150/ijbs.58422 (PMC8193262; doi:10.7150/ijbs.58422)
Supplement: Supplementary file 1 — Supplementary figures and tables. [file ijbsv17p1979s1.pdf]

Supplementary Table and Figures

Table S1. Primer sequences used for qRT-PCR.

**Primers for qRT-PCR**		
Gene name	Forward Primer	Reverse Primer
GAPDH	AAGGTCGGAGTCAACGGATTTG	CCATGGGTGGAATCATATTGGAA
18S rRNA	GTAACCCGTTGAACCCCATT	CCATCCAATCGGTAGTAGCG
U6	CTCGCTTCGGCAGCACA	AACGCTTCACGAATTTGCGT
β-actin	TCGTGCGTGACATTAAGGAG	ATGCCAGGGTACATGGTGGT
SH3PXD2A-AS1	CCACATAGGTCTCACTTA	AGGTAATTCTTCTCTGATTG
CD82	GCTCATTCGAGACTACAACAGC	GTGACCTCAGGGCGATTCA
CX3CL1	ACCACGGTGTGACGAAATG	TGTTGATAGTGGATGAGCAAAGC
GML	GTGAGGCTGGGGGTATCAAAA	GCAACATTTCTCTGCGGAACA
DR5	GGAGGATTGCGTTGACGAGA	GGTGTATTTTGTGGGCGCAG
BDKRB2	CCGAAAGAAGTCTTGGGAGGT	CTGGCGTTCCACGGAGATG
ANLN	ATCTTGCTGCAACTATTTGCTCC	TCCTGCTTAACACTGCTGCTA
FAS	TCTGGTTCTTACGTCTGTTGC	CTGTGCAGTCCCTAGCTTTCC
TP53AIP1	CTGGCTGGGTTTCAGATCCC	CAGTGGCCTGTCTCTAAGCA
S100A2	GCCAAGAGGGCGACAAGTT	AGGAAAACAGCATACTCCTGGA
TGFA	AGGTCCGAAAACACTGTGAGT	AGCAAGCGGTTCTTCCCTTC
SCD	GCCCCTCTACTTGGAAGACGA	AAGTGATCCCATACAGGGCTC
SFN	TGACGACAAGAAGCGCATCAT	GTAGTGGAAGACGGAAAAGTTCA
PAI1	AAAGGCAACATGACCAGGCT	ATGCGGGCTGAGACTATGAC
MET	AGCAATGGGGAGTGTAAAGAGG	CCCAGTCTTGTACTCAGCAAC
GDF15	ACCTGCACCTGCGTATCTCT	CGGACGAAGATTCTGCCAG
IER3	CAGCCGCAGGGTTCTCTAC	GATCTGGCAGAAGACGATGGT
TRPM2	TCCCCGCCGAGTACATACTG	GTCTGCTCCGATATGAACTTCTC
PIG3	GGCACAGCTGCTATCCAACT	AGCATTTGCTTGTACTTTGGTGA
PCNA	TTGCACGTATATGCCGAGACC	GGTGAACAGGCTCATTCATCTCT
EGFR	TTGCCGCAAAGTGTGTAACG	GTCACCCCTAAATGCCACCG
MDM2	GGCAGGGGAGAGTGATACAGA	GAAGCCAATTCTCACGAAGGG
P2RX6	GAACCCCAGTTTTCCATCATCA	GGCGTCACAAGGAAGTTGGT
CDC25C	ATGACAATGGAAACTTGGTGGAC	GGAGCGATATAGGCCACTTCTG
SLC38A2	ACCGCAGCCGTAGAAGAATG	GCCAGACGGACAATGAGAAGAA
GADD45A	GAGAGCAGAAGACCGAAAGGA	CAGTGATCGTGCGCTGACT
RRM2B	ATTGGGCCTTGCGATGGATAG	GAGTCCTGGCATAAGACCTCT
**Primers for CHIP assays**		
Gene name	Forward Primer	Reverse Primer
CD82	ACTGGTTGTTCTGGGCTACTTC	TGAATCTCATGGAGGCGGG
CX3CL1	TGCTGGCCTTTTGTGTGTTG	CAAGCTGGGAACATGCCCTA
DR5	ACACATAAATCAGCACGCGG	GTAGATCGGGCATCGTCGG
IER3	TGTGAGGGATCCTGTGGCTA	CTGCACGTTGTGAGTGTGTG

SCD	CGGGACGGAGATGTTAGTGG	TCGGGAGCTTTCTCTCTGGA
MET	CGATTTCCCTCTGGGTGGTG	CCCAGCACGTGTCTGTCTG
PCNA	ATAAAGCTGGGGCTTGACGA	CCGCCTCTTTGACTCCTGAA
SFN	AGACACAGAGTCCGGCATTG	CAGCTTGGCCTTCTGGATCA
GDF15	CAGCATCTGGTCAGTCCCAG	TCACCGTCCTGAGTTCTTGC
TGFA	GACGGTAGCCGCCTTCCTATT	GTGCGGGGGAAAAAGACG

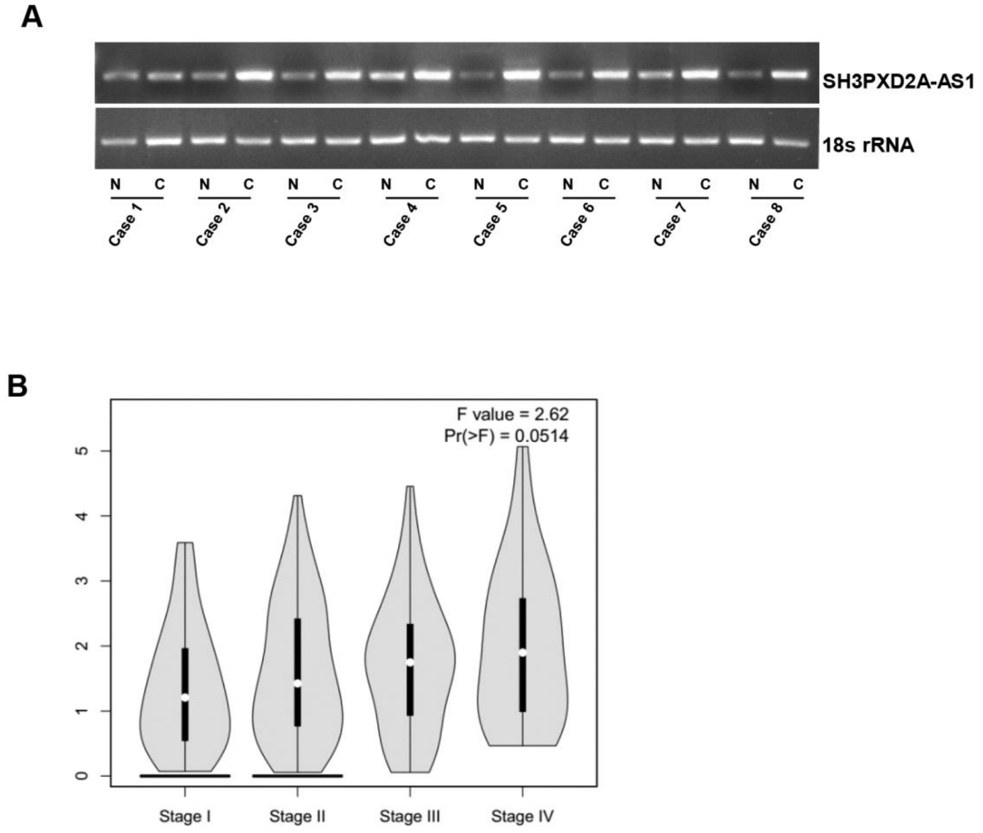

Figure S1. SH3PXD2A-AS1 is overexpressed in CRC tissues. **(A)** SH3PXD2A-AS1 is upregulated in CRC tissues detected by RT-PCR running on gel electrophoresis by using paired colon tissues. **(B)** SH3PXD2A-AS1 expression in different stages of CRC in TCGA database analyzed in the GEPIA website (http://gepia.cancer-pku.cn/index.html).

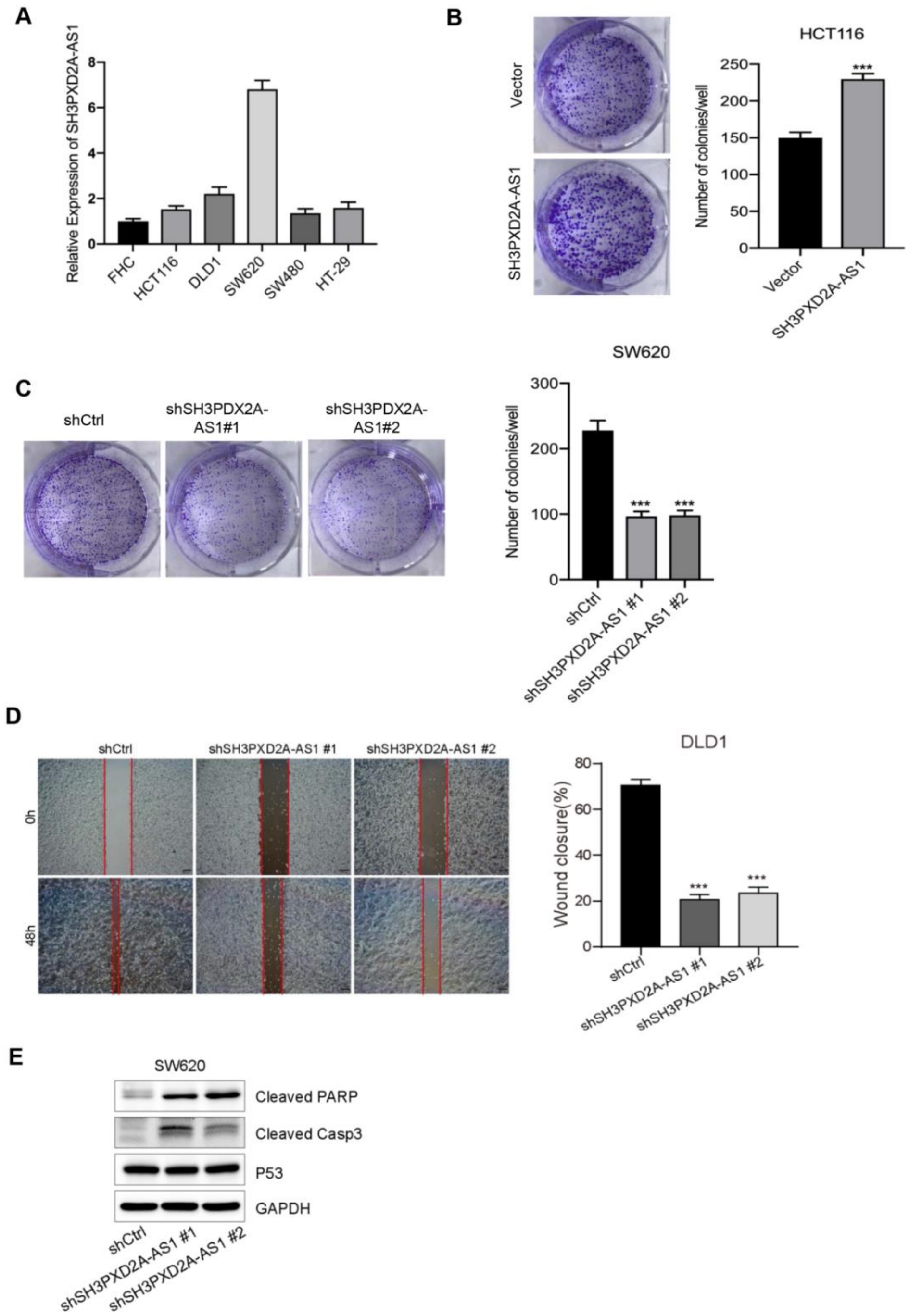

Figure S2. SH3PXD2A-AS1 promotes CRC proliferation and migration. **(A)** SH3PXD2A-AS1 expression was detected in CRC cell lines and the normal colon cell line, FHC. **(B-C)** SH3PXD2A-AS1 promotes CRC cells colony formation. **(D)** SH3PXD2A-AS1 Knockdown inhibits wound healing in DLD1 cell. **(E)** SH3PXD2A-AS1 knockdown increases the expression of cleaved PARP and cleaved casp3. ***p < 0.001 by two-tailed Student's t test.

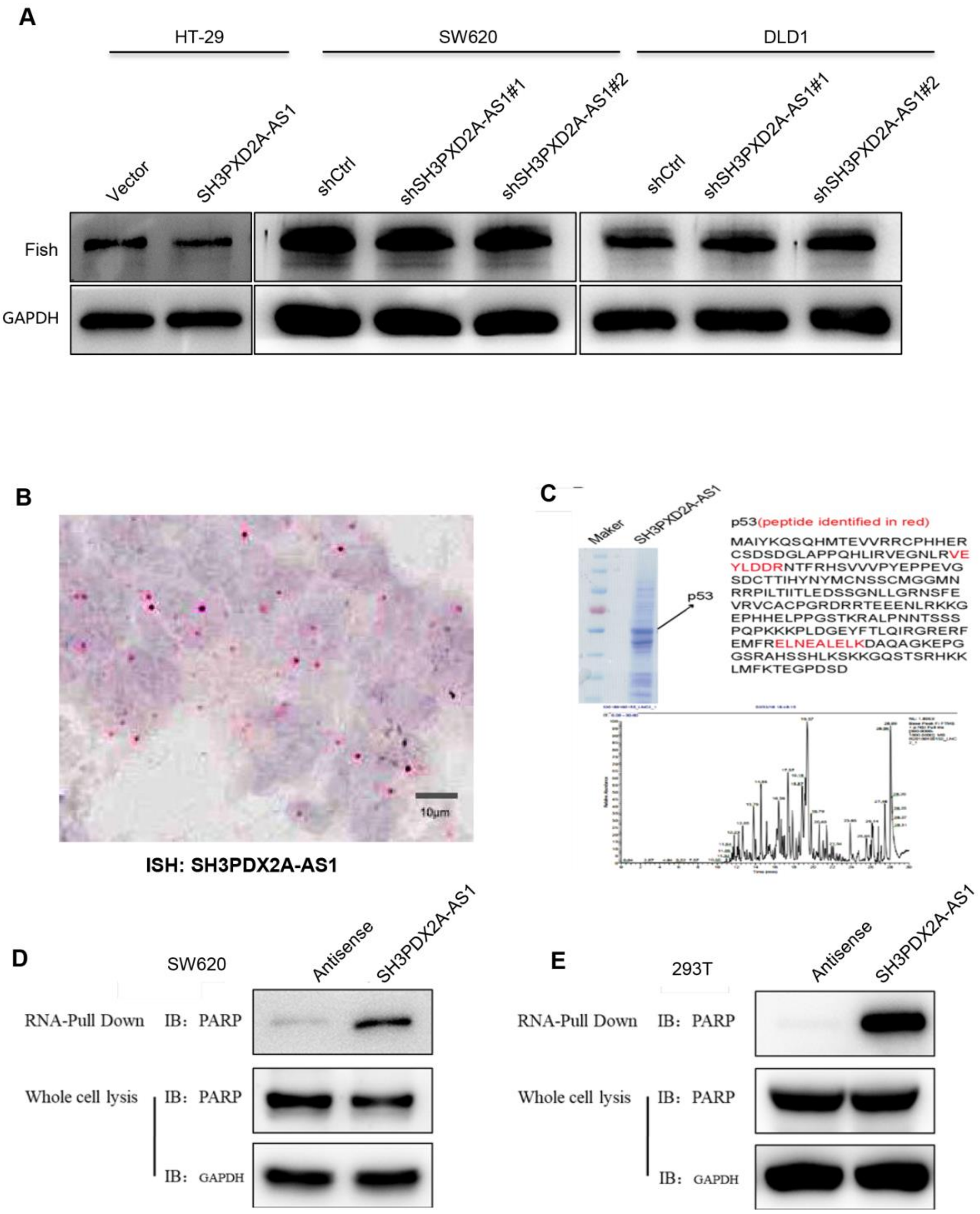

Figure S3. SH3PXD2A-AS1 can also interact with PARP. **(A)** SH3PDX2A-AS1 did not affect the protein expressions of the neighbor gene, Fish **(B)** RNA-ISH showed SH3PXD2A-AS1 located both in the nuclei and the cytoplasm CRC tissues. **(C)** Mass spectrum analysis of SH3PXD2A-AS1 binding proteins. **(D-E)** Biotin-RNA pull-downs followed by Western-blot by using Anti-PARP in SW620 and 293T cells.

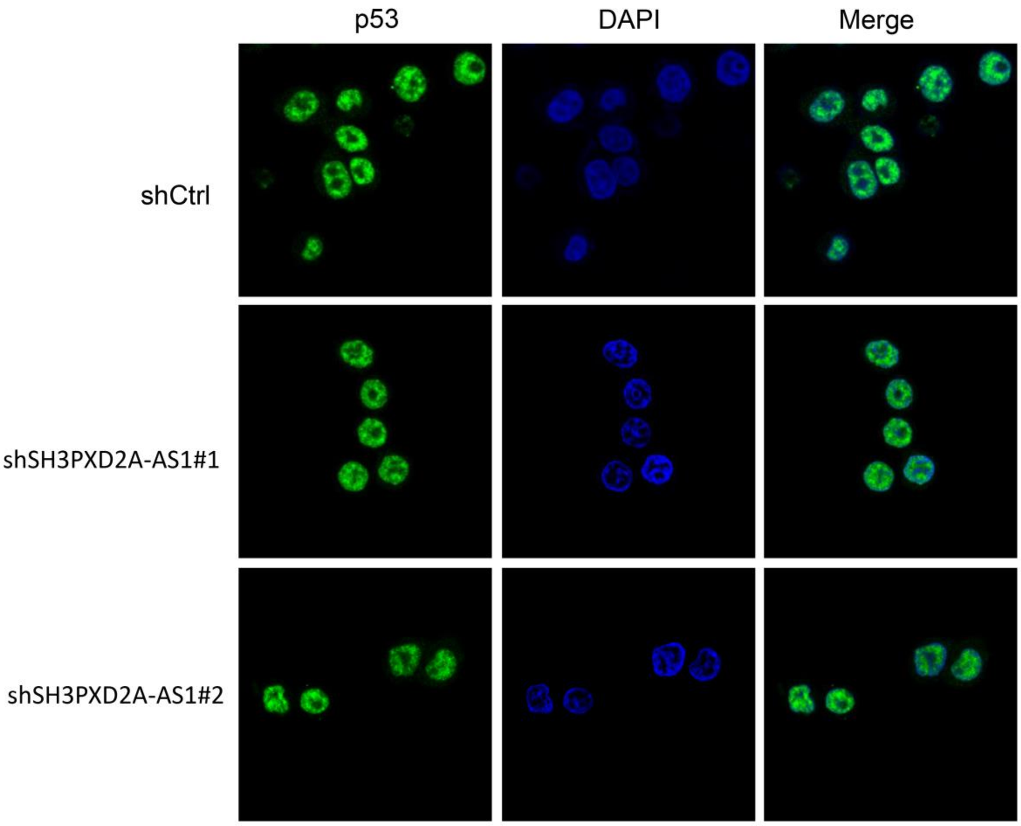

Figure S4. SH3PXD2A-AS1 Knockdown did not affect the distribution of p53 in nucleus or cytoplasm. Immunofluorescence of p53 in shCtrl、shSH3PXD2A-AS1#1 and SH3PXD2A-AS1#2 SW620 cells.

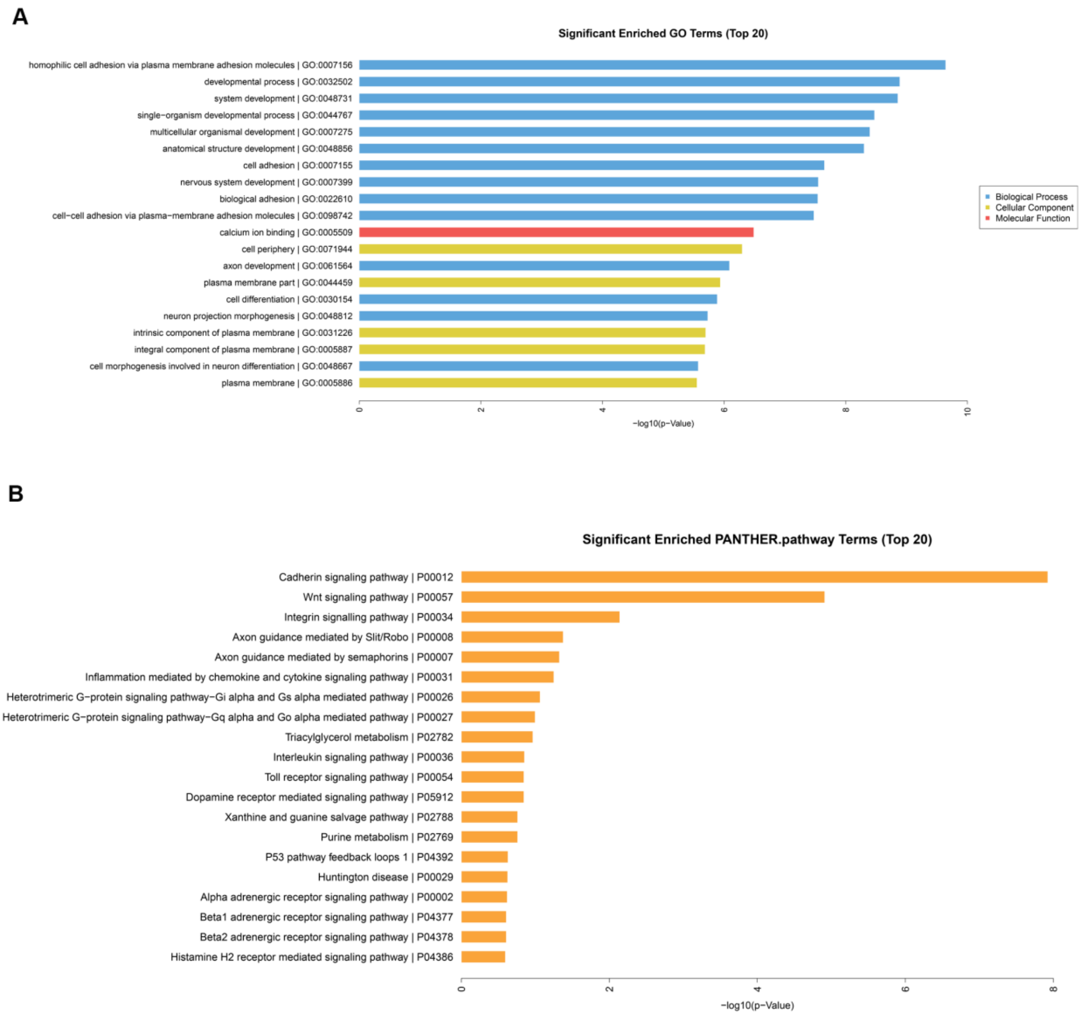

Figure S5. Gene ontology analysis of SH3PXD2A-AS1 related pathways by using RNA-seq data. (A-B) Differentially expressed genes defined by functionally enriched gene ontology (GO) terms and pathways involved in cadherin signaling pathway, Wnt signaling pathway and P53 pathway

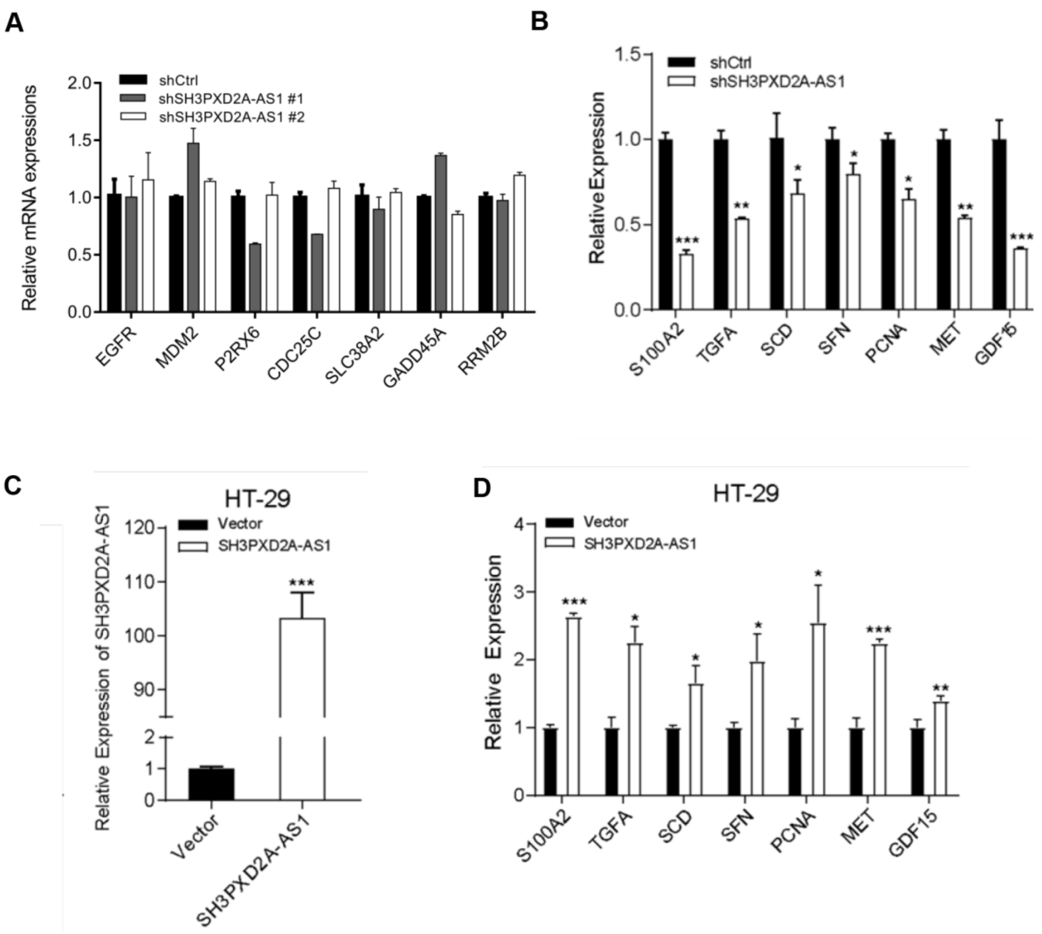

Figure S6. SH3PXD2A-AS1 regulates p53 target genes. (A) Relative mRNA expressions of p53 targets those not changed in SH3PXD2A-AS1 knockdown SW620 cells. (B) Relative mRNA levels of several p53 targets in xenograft tumors. (C-D) Relative mRNA levels of several p53 targets in SH3PXD2A-AS1 overexpression HT29 cells.

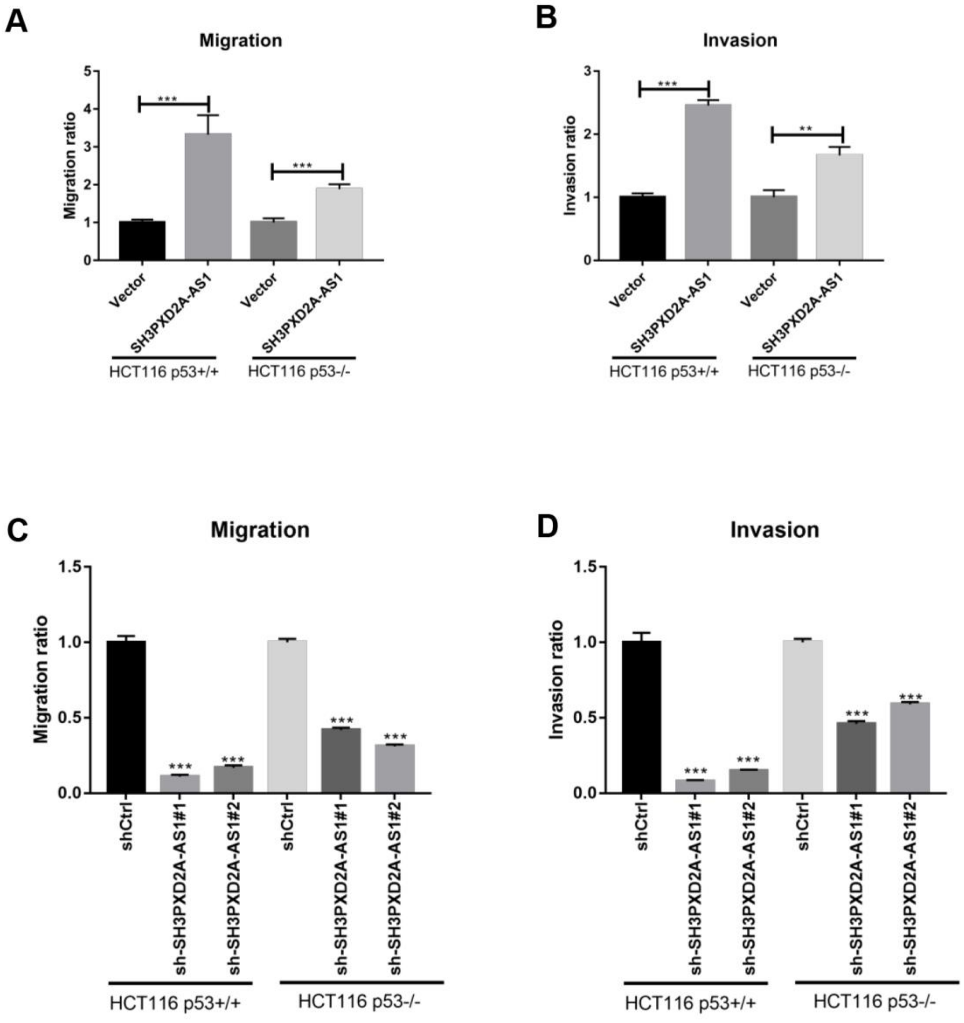

Figure S7. Effects of SH3PXD2A-AS1 on cell migration and invasion in $p53^{-/-}$ HCT116 cells. **(A-D)** Effects of SH3PXD2A-AS1 overexpression or knockdown on cell migration and invasion in $p53^{-/-}$ HCT116 cells. $^{**}p<0.01$ and $^{***}p < 0.001$ by two-tailed Student's t test.

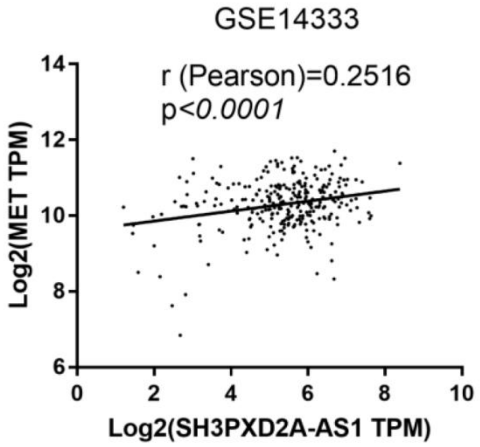

Figure S8. Correlation analysis of relative mRNA levels of SH3PXD2A-AS1 and MET by using the GEO dataset (GSE14333).
